# Polarisation independent silicon-on-insulator slot waveguides

**DOI:** 10.1038/srep37760

**Published:** 2016-11-29

**Authors:** Valerian Hongjie Chen, Jun Rong Ong, Ching Eng Png

**Affiliations:** 1 Institute of High Performance Computing, 1 Fusionopolis Way, 16-16 Connexis, 138632, Singapore

## Abstract

The minimisation of birefringence, or polarisation mode dispersion, is vital for simplifying and miniaturising photonic components. In this work, we present a systematic study of the slot waveguide geometries required for having zero birefringence (ZB). We show that the rail widths required for ZB are more strongly dependent on the height of the waveguide than on the slot separation. After which, we demonstrate that the ZB geometry is significantly affected by the slanting of the waveguide walls. This paper proceeds to show that within the range studied, one can fix the height, slot, slant angle, and bend radius, and still achieve ZB by varying the widths of both of the rails. Given a fabrication tolerance of 5 nm, we show that a coherence length on the order of a hundred microns can be achieved. We finish by showing that for straight and bent ZB waveguides, having symmetric rails is preferable due to higher tolerances and lower sensitivity to bending. Since any arbitrarily shaped slot waveguide is a combination of both single mode straight and bent waveguides, we have a toolbox from which we can achieve ZB for any given slot and height.

Slot waveguides enable large fields to be confined in the low index material^1^,^2^, enabling a variety of applications such as nonlinear optics^3^, all-optical logic gates^4^, electro-optical modulators^5^, and optical sensors for chemical and biological applications^5^,^6^,^7^. In the single mode regime, such waveguides support a quasi-transverse electric (TE) and quasi-transverse magnetic (TM) mode. Due to the subwavelength nature of these waveguides, the modes are neither entirely TE nor TM. However, since they are still largely TE or TM, we will drop the ‘quasi’ prefix for the rest of the paper. These TE and TM modes usually have different effective refractive indices, leading to a varying phase between them. Since slot waveguides are often used for phase sensitive devices like sensors, the birefringence of the TE and TM modes poses a problem. Consequently, a lot of effort has been spent on splitters to separate the TE and TM modes so that they can be processed separately^8^. The possibility of achieving low birefringence in slot waveguides has been noted in previous work on polarisation management^8^. It was deemed to be a nuisance, since it makes separating the modes more difficult.

However, if we could eliminate the birefringence of the TE and TM modes, we would not need to separate the TE and TM modes in the first place. Hence, instead of having two sets of components, one for the TE mode and one for the TM mode, one set of zero birefringence (ZB) components would suffice. Consequently, the footprint of such photonic circuits may be reduced by as much as half. This will not only engender reductions in cost but will also facilitate the fabrication of small photonic chips where real estate is of paramount importance. Zero-birefringence rib^9^,^10^,^11^, strip^12^,^13^,^14^, and annular photonic crystal^15^ waveguides and their applications have been previously studied to a reasonable level of detail. Although ZB slot waveguides have been studied in specific applications including couplers^3^,^16^, strip-slot converters^17^ and slot microring resonators^18^, to the best of the authors’ knowledge, there has yet to be an extensive systematic study of the geometries required to achieve ZB in slot waveguides.

We begin our study by considering a coherence length *l*_*c*_ = *λ*/2Δ*n*_eff_, the length over which the TE and TM mode acquires a phase of *π* with respect to each other. This is dependent on the difference in effective refractive indices of the TE and TM modes (Δ*n*_eff_ = *n*_eff, TE_ − *n*_eff, TM_). It is, strictly speaking, incorrect to take the effective refractive index to be the weighted average of the waveguide materials’ refractive indices^19^. However, we can use effective medium theory as an approximation^20^ — the effective refractive index can thus be thought of as the weighted average of the refractive indices of silicon and silicon dioxide, with the magnitude of the E field in either material determining the relative weights. The weight for the refractive index of silicon is the core confinement, which is given by


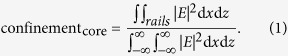


Assuming we are operating at telecommunications wavelengths, should we have Δ*n*_eff_ < 0.0005, we can have *l*_*c*_ in the millimetre scale, which is large compared to the size of typical photonic components. For practical purposes, we shall therefore consider the birefringence to be zero if the effective refractive index Δ*n*_eff_ less than 0.0005; that is, the birefringece is zero to three decimal places.

## Results

### Simulation set-up

**Figure 1:**
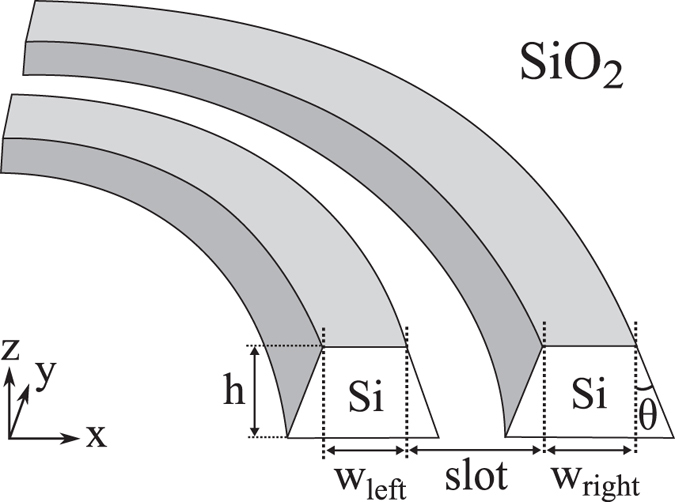
Si-SiO_2_ slot waveguide geometry; the rails are made of Si while the substrate and BOX are made of SiO_2_. The geometric parameters are the height *h*, widths of the rails *w*_left_ and *w*_right_, slot width *slot*, slant angle *θ*, and the bending radius (radius of curvature). For waveguides with slanted walls, the slot was measured between the top of the rails, that is, where the separation between the waveguides is largest. For bent waveguides, the bending radius was taken to be the distance between the centre of curvature and the middle of the slot.

The cladding and buried oxide (BOX) were taken to be made of SiO_2_ while the waveguide cores were taken to be made of Si. It is important to note that the core is embedded entirely in the SiO_2_ cladding and BOX; there is no air in contact with the cladding at all (Fig. 1). This particular combination of materials was chosen for its simplicity and symmetry, a good starting point to understand zero-birefringence. The geometric parameters are defined in Fig. 1. In this work, unless it is stated otherwise, the bending radius is infinite (straight waveguides) and the slant angle *θ* is zero (straight walls).

A wavelength of 1550 nm, a typical wavelength for telecommunications, was used for all simulations. The wavelength was not varied since this paper is concerned with how the waveguide geometry affects the modal dispersion; investigating the chromatic dispersion is a beyond the scope of this paper.

The height, slot, and slant angle *θ* of the waveguides were then varied separately, and the rail widths required for ZB were determined. We chose to study asymmetric waveguides, in that the width of the left and right rails are not the same. By allowing for such asymmetry, unlike a previous study^18^, a greater flexibility of the choice of the other geometric parameters may be achieved. More importantly, such asymmetry is required for making slot waveguides with sharp bends^21^.

The simulations were then carried out in a range of geometries where only two modes, one TE and one TM, are supported. This set some constraints on the geometric variables *slot*, *w*_left_, and *w*_right_. When these parameters are too large, the waveguide becomes multi-modal^22^. Since the higher order modes tend to have a significantly different effective refractive index than the first order modes, zero-birefringence would be harder, if not impossible, to achieve. When these parameters are too small, the modes are poorly confined and thus have higher attenuation due to leakage loss. When the confinement is especially poor, the field at the boundaries of the simulation region is no longer negligible, and this leads to unphysical modes which we have to rule out^23^. The slot confinement, or the fraction of the magnitude of the E field in the slot, is given by Equation 2,


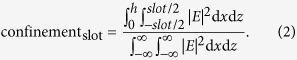


We neglect any ZB geometries where the slot confinement of the TE mode is less than 0.05, which we consider to be too low, leading to the problems highlighted in the previous paragraph. These considerations of how large and small the parameters should be motivated our choice of the waveguide geometries to study.

**Figure 2:**
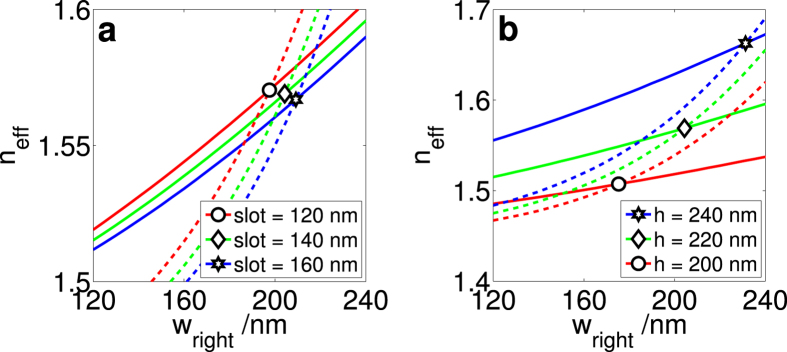
The effective refractive index of the TE and TM modes. The dashed lines denote TE modes while the solid lines denote TM modes. The points at which the corresponding solid and dashed lines cross are marked with various shapes, giving the value of *w*_right_ at which there is no difference in the effective refractive index, that is, there is zero-birefringence. For both figures, the width of the left rail *w*_left_ is fixed at 150 nm while the width of the right rail *w*_right_ is varied. The waveguides were taken to be straight and to have vertical walls. (**a**) Changing the size of the slot while the height is fixed at 220 nm and (**b**) changing the height while the slot is fixed at 140 nm. The corresponding effective refractive indices of the modes are calculated. We found that such behaviour is typical for all slot waveguides we studied which have one TE and one TM mode.

The effective refractive indices of the TE (dashed lines) and TM (dotted lines) modes of straight waveguides were calculated as a function of *w*_right_ (Fig. 2). The other geometric variables were fixed at *w*_left_ = 150 nm and *θ* = 0°. In Fig. 2a, the height is set to 200 nm; in Fig. 2b, the slot is fixed at 140 nm. In general, we see that when *w*_right_ is narrower, the TE mode has a lower effective index than the TM mode. However, as *w*_right_ is increased, the effective index of the TE mode increases much more quickly than the TM mode. Hence, their effective indices are the same at a particular geometry, which is the value of *w*_right_ at which the two lines cross each other. This behaviour is typical of slot waveguides with one TE and one TM mode. In fact, in all the geometries studied, as long as the slot waveguide remains single mode, a geometry can always be found to satisfy the zero-birefringence condition.

The mode profiles of the TE and TM modes at ZB are not particularly elucidating. This is in contrast to strip waveguides, where ZB is achieved when the waveguide is square - the symmetry leads to the degeneracy of the TE and TM modes. Consequently, the mode profiles are not shown here; typical slot waveguide mode profiles may be easily found elsewhere (for example ref. 16). In general, the field of the TE mode is confined in the slot, whereas the field for the TM mode is located mainly above and below the rails.

**Figure 3:**
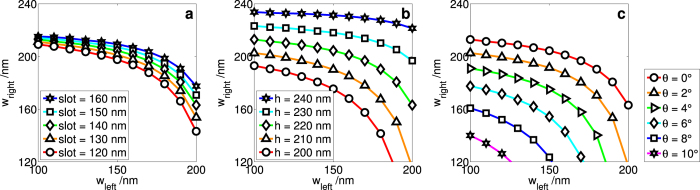
The dimensions of the left and right rails required for achieving ZB at various geometries. Varying (**a**) the slot (*h* = 220 nm and *θ* = 0°), (**b**) the waveguide height (*slot* = 140 nm and *θ* = 0°), and (**c**) the slant angle *θ* (*slot* = 140 nm and *h* = 220 nm). For ease of comparison, note that the same geometry is denoted by the green line with diamonds in (**a**) and (**b**), and the red lines with circles in (**c**).

Here, it is not immediately obvious which geometries will lead to zero-birefringence. Consequently, we find the zero-birefringence geometry using the bisection method (Fig. 3) and the interpolation of parameter sweeps (subsection on bent waveguids) for a variety of slots, heights, slant angles *θ*, and bending radius, which will help to shed light on this matter.

### Effect of slot, height, and slant angle *θ* on ZB geometry

The first interesting observation is that to achieve zero-birefringence for given a certain slot, height, and slant angle *θ*, the greater the width of one of the rails, the smaller the width of the other (Fig. 3a–c). Since the effective refractive index is a result of the mode seeing some of the core (that is, the rails) and some of the cladding, the core confinement (the fraction of the E field in the rails) gives a good understanding of what is happening to the effective refractive index. We found that the larger either of the rails are, the greater the core confinement, which is key to understanding how the widths of the rails required for ZB are related.

Consider a geometry where there is no birefringence. We then increase the width of the left rail. The confinement in the core, and hence the effective refractive index, for increases for both the TE and TM modes. However, this increase is different for each of the modes, hence the difference in effective refractive indices is now non-zero. The width of the right rail must be decreased to decrease the core confinement, and hence counteract the effect of increase in confinement due to having a larger left rail. Consequently, we see that having asymmetric rail widths allows for much greater control over the confinement, and thus the effective refractive indices. This allows us to achieve zero-birefringence in a greater range of geometries, which will be explored in the following paragraphs.

The greater the slot size, the wider the rails need to be to achieve ZB (Fig. 3a). Similarly, the greater the waveguide height, the wider the rails need to be (Fig. 3b) to achieve ZB. The reason is as follows. When the slot or the height is increased, the effective refractive index of the modes is decreased (slot) and increased (height) respectively (Fig. 2). This is as expected. With a wider slot, there is less overlap of the evanescent field of a given rail with the other rail, hence there is a weaker field in the high index region. With a greater height, the rails are larger, hence there is a stronger field in the high index region. Consequently, effective medium theory explains why the effective refractive index is smaller with a wider slot and larger with a greater height.

However, these two changes in confinement have the same effect on the widths of the right rails required in order to achieve ZB: the rails need to be wider (Fig. 2). It is also worth noting that the widths of the rails required for zero-birefringence are much more sensitive to the waveguide height than they are to changing the slot. This is largely a consequence of the effective refractive indices *n*_*eff*_ being much more sensitive to the height than the slot separation. A deeper understanding of the physics behind these phenomena may be acquired by considering the decay lengths of the evanescent E-field outside the rails^16^ (in the paper cited, the decay length is called the coupling length), since the effective refractive index is simply a reflection of how much of the E-field is confined within the rails and how much of it is outside.

Increasing the slant angle *θ* has a similar effect on *w*_left_ and *w*_right_ decreases the rail widths required to achieve ZB. A maximum slant angle of *θ* = 10° was considered, since that was already achievable by fabrication techniques several years ago^24^. Qualitatively speaking, given the definition of the geometric parameters used in this paper, this is to be expected. Slanting the walls can be thought of as effectively increasing the rail widths and decreasing the slot size. A more rigorous approach to incorporating the slant angle as the effective parameters of a straight-wall waveguide can be found in this paper^25^.

### Tolerance

**Figure 4:**
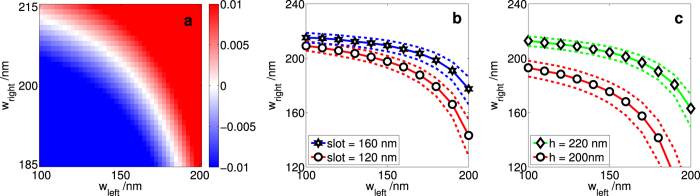
Understanding the tolerance of ZB slot waveguides. (**a**) How the difference in the effective refractive indices of a straight slot waveguide Δ*n*_eff_ = *n*_eff, TE_ − *n*_eff, TM_ is affected when *w*_left_ and *w*_right_ are varied; *slot* = 140 nm, *h* = 220 nm, and *θ* = 0°. The colour bar shows Δ*n*_eff_ for a given combination of *w*_left_ and *w*_right_. In (**b**) and (**c**), the solid lines show Δ*n*_eff_ = 0.0005 and the dashed lines show Δ*n*_eff_ =  ± 0.005; the other parameters which were not varied are kept at the same value as that in (**a**). (**b**) The slot size has a negligible effect on tolerance. (**c**) The tolerance is relaxed when the height is reduced.

The polarisation mode dispersion is very sensitive to the widths of the rails. An example of the typical tolerance is shown in Fig. 4a. The rail widths required to achieve ZB have to be accurate to roughly a nanometre. Consequently, although it may currently not be possible to reliably fabricate structures with Δ*n*_eff_ < 0.0005, it may still be feasible to fabricate structures where the requirements on Δ*n*_*eff*_ are less stringent. For example, if we ease the requirement for *l*_*c*_ by an order of magnitude, that is, by requiring coherence over hundreds of microns rather than over millimetres, then we need Δ*n*_eff_ < 0.005. As we can see from the figure, this requires a fabrication tolerance of around ±5 nm. Considering that wider features could be fabricated with a tolerance of ±7 nm a few years ago^26^, it is reasonable to expect that ±5 nm is achievable with current technology.

The tolerance of either rail depends on their widths (Fig. 4a–c). Assuming that the fabrication tolerance over this range of widths is roughly the same, then the rails should be designed to be symmetric (*w*_left_ = *w*_right_), such that neither rail’s tolerance is a limiting factor. Interestingly, in the range of geometries studied, we see that the slot has a negligible effect on the tolerance (Fig. 4b), but having a smaller height increases the tolerance (Fig. 4c).

### Bent waveguides

**Figure 5:**
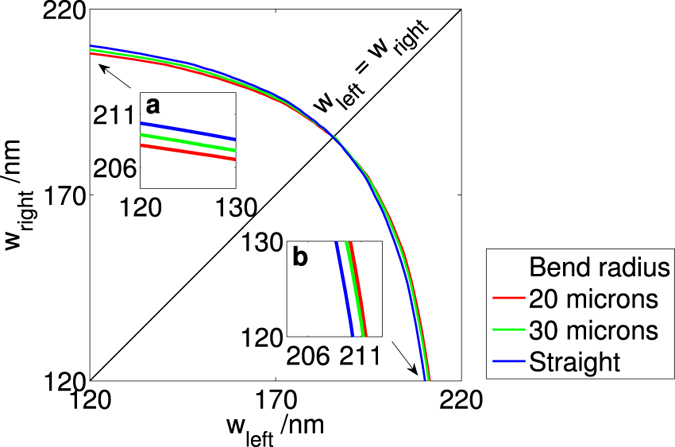
The effect of the bend radius on the ZB geometry, for *slot* = 140 nm, h = 220 nm. The black line shows *w*_left_ = *w*_right_. The bend radius is measured from the centre of the slot. The centre of the bend is in the negative x direction, that is, to the ‘left’ of the left rail. In other words, the left rail is the inner rail and the right rail is the outer rail. Below a bending radius of 20 nm, confinement is less than 0.05 for most combinations of rail widths studied, and hence not shown here. Note that when the outer rail is wider than the inner rail, a larger bending radius leads to requiring a slightly narrower outer rail for ZB. This effect is reversed when the inner rail is wider. Insets (**a**) and (**b**) are magnified sections of the graph where the rails are asymmetric.

In order to further the understanding of ZB slot waveguides, we studied what would happen if the waveguides were bent and not straight. We chose a slot size of 140 nm, height of 220 nm, width of the rails between 100 nm and 240 nm, and swept the bending radius from 10 *μ*m to 100 *μ*m (Fig. 5). We found that above 60 *μ*m, the effect of the bending is negligible. At 10 *μ*m, the mode is poorly confined (core confinement of less that 5 percent) at most of the rail widths, hence we did not study waveguides with a larger curvature than that. At a bending radius of 20 *μ*m, the adjustment of the rail widths required to account for the bend is at most 3 nm, and this difference is smaller when the waveguide widths are more symmetric. When the outer rail (right rail) is larger than the inner rail (left rail), a larger bending radius requires a smaller outer rail (Fig. 5 inset a). This effect is reversed when the outer rail is smaller than the inner rail (Fig. 5 inset b).

Hence, for phase sensitive applications, we propose that the curvature can be increased from 0 to the desired value linearly (that is, shaped like an Euler spiral), and the widths of the rails can be tapered to the appropriate value, while keeping the height and the slot fixed. It is of greater interest to determine the widths of the rails at which ZB is maintained — even when the bending radius is changed — removing the need for such tapering. Incidentally, this occurs when the rails are symmetric, that is when *w*_left_ = *w*_right_.

With bent and straight waveguides, waveguides of arbitrary shapes can be constructed (assuming that overlap mismatches do not cause significant birefringence). It is unfortunate that in the range of geometries studied, we were unable to achieve ZB and the low bending losses reported in another study^21^ simultaneously. Achieving ZB in tight bends with low losses may be an interesting topic for a future study.

## Discussion

In order to further miniaturise photonic circuits and integrate them on a large scale, it is important to minimise the crosstalk between the various components. Recent breakthroughs in extreme skin depth (e-skid) waveguides address this challenge by exploiting anisotropic materials to suppress the evanescent field outside the waveguides without using metals, which are lossy^27^,^28^,^29^. Due to the importance of minimising crosstalk, studying how to achieve ZB in e-skid strip waveguides would be an appropriate next step. Moreover, since the effective refractive index is approximately a result of effective medium theory, having a greater control of the evanescent fields could lead to better tunability of the effective refractive index, which may help achieve ZB more reliably over a wider range of geometries and wavelengths. These considerations would need to take into account the types of anisotropic metamaterials that are practical to fabricate as the cladding. Slot waveguides are already more difficult to fabricate than strip waveguides; applying e-skid engineering while achieving ZB and maintaining their utility (for example, as sensors or for non-linearity), in the opinion of the authors, is a complex but desirable endeavour which is still several steps from fruition.

In this work, we have shown that it is possible to design slot waveguides with negligible differences in the effective refractive index of the TE and TM modes. However, such waveguides are very sensitive to the geometry and hence difficult to fabricate reliably. Nonetheless, depending on the coherence length required, it is still possible and desirable to fabricate such waveguides. We found that in order to better achieve ZB, the slot width is not particularly important but having a smaller height is preferable since such geometries relax the tolerance. It is important to consider the slanting of the walls of the rails since this has a significant impact on the ZB geometry. We have also studied bent waveguides, and have shown that the bending only marginally affects the ZB geometry, even for bend radii down to 20 *μ*m. When the bend radius is too small, the mode is poorly confined — this turns out to be the main issue with bent waveguides, while changes in the ZB cross-sectional geometry arising from bending are much less problematic. Interestingly, we found that for both straight and bent waveguides, having symmetric rail widths is preferable. With the understanding of straight and bent slot waveguides presented here, slot waveguide components of arbitrary geometry can be designed as a combination of individual straight and bent waveguides.

## Methods

### Material data

The material properties were taken from a Sellmeier fit of the optical data from Malitson (SiO_2_)^30^ and Salzberg and Villa (Si)^31^. Since we used a wavelength of 1550 nm, this corresponds to having 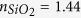 and *n*_*Si*_ = 3.48 (to three significant figures).

### Eigenmode calculation

Lumerical MODE Solutions^32^ was used to calculate the eigenmodes of the various slot waveguides. The simulation area was 2 *μ*m by 2 *μ*m. Since the field intensity of physical modes is small when far from the waveguide, metallic boundary conditions (BCs) were used instead of perfectly matched layer BCs to speed up the simulations. As a sanity check, we used PML BCs for several simulations, and found that they were in good agreement with simulations where metallic BCs were used. Modes with an effective refractive index less than the cladding were considered to be unphysical and thus removed^23^.

Given the length scales involved in the simulation, the mesh size at the center of the simulation region, where the rails and slot are, was chosen with the following considerations. The height was the only length parameter in the z direction. Since we varied the height at intervals of at least 10 nm, a mesh size of 10 nm was used along the z-axis. However, since the width of the right rail was optimised to a high precision, a smaller mesh size was used. We found that there was no noticeable difference in the ZB geometry when the mesh size was varied from 5 nm to 1 nm, hence a mesh size of 5 nm was used along the x-axis.

### Optimisation

For particular values of a certain slot, height, and width of the left rail, the bisection method was used to find the width of the right rail such that the |Δ*n*_eff_| would be minimised. We chose the bisection method because it enabled reliable convergence. For bent waveguides, there were several minima of |Δ*n*_eff_| (that is, there was more than one possible width of the right rail), hence a different method was used. A parameter sweep was performed, and the results were interpolated with Matlab in order to find the optimal ZB geometries. We then neglected the ZB geometries where the TE slot confinement was less than 0.05.

## Additional Information

**How to cite this article**: Chen, V. H. *et al*. Polarisation independent silicon-on-insulator slot waveguides. *Sci. Rep*. **6**, 37760; doi: 10.1038/srep37760 (2016).

**Publisher's note:** Springer Nature remains neutral with regard to jurisdictional claims in published maps and institutional affiliations.
